# ﻿Revision of the orb-weaving spider genus *Yaginumia* Archer, 1960 (Araneae, Araneidae) from China

**DOI:** 10.3897/zookeys.1223.139465

**Published:** 2025-01-06

**Authors:** Xiaoqi Mi, Cheng Wang, Ming Su

**Affiliations:** 1 College of Agriculture and Forestry Engineering and Planning, Guizhou Provincial Key Laboratory of Biodiversity Conservation and Utilization in the Fanjing Mountain Region, Tongren University, Tongren 554300, Guizhou, China Tongren University Tongren China; 2 Central South Inventory and Planning Institute of National Forestry and Grassland Administration, Changsha 410007, Hunan, China Central South Inventory and Planning Institute of National Forestry and Grassland Administration Changsha China

**Keywords:** Arachnida, diagnosis, identification key, morphology, new species, taxonomy

## Abstract

The orb-weaver spider genus *Yaginumia* Archer, 1960 from China is revised, and three species, including two new species, are recognized: *Y.medog* Mi & Wang, **sp. nov.** (♂♀) from Xizang and *Y.qiong* Mi & Wang, **sp. nov.** (♂♀) from Hainan; the type species, *Y.sia* (Strand, 1906) (♂♀), is redescribed based on specimens from Guizhou and Hubei. A distributional map of the studied specimens is also provided.

## ﻿Introduction

The orb-weaver spider genus *Yaginumia* Archer, 1960 was established by Archer (1960) to accommodate the type species *Y.sia* (Strand, 1906). This species was first described as a member of the genus Araneus Clerck, 1757 under the subgenus Zilla C.L. Koch, 1834 (Bösenberg and Strand 1906); then it was transferred to the genus *Zygiella* F.O. Pickard-Cambridge, 1902 by Roewer (1942) without any justification; Saitō (1959) returned it to the genus *Zilla*, again without justification. Levi (1974) treated it in *Zygiella*, but pointed to two characters that are unusual for typical *Zygiella* species, such as the wider spacing of the eyes and the cap on the embolus. Consequently, most subsequent taxonomic studies placed this species in the genus *Yaginumia* (WSC 2024). Several studies using traditional Sanger sequencing revealed that this genus was closely related to the genus *Guizygiella* Zhu, Kim & Song, 1997 (Gregorič et al. 2015; Kallal and Hormiga 2018; Kallal et al. 2020).

*Yaginumiasia* (Strand, 1906) always lives close to humans in and around houses, other buildings, rice fields and cotton fields. It is widely distributed in China (Lee 1966; Yin 1978; Song 1980; Hu 1984; Guo 1985; Zhang 1987; Chen and Gao 1990; Feng 1990; Chen and Zhang 1991; Zhao 1993, 1995; Yin et al. 1997, 2012; Song et al. 1999; Zhu and Zhang 2011), Japan (Bösenberg and Strand 1906; Roewer 1942; Saitō 1959; Archer 1960; Yaginuma 1960, 1971, 1986; Levi 1974; Ishinoda 1989; Baba and Tanikawa 2015; Tanikawa 2007; 2009) and Korea (Namkung et al. 1972; Chikuni 1989; Namkung 2002, 2003; Kim and Cho 2002; Kim and Kim 2002; Kim and Lee 2012).

*Yaginumia* specimens deposited in the Museum of Tongren University were examined, and three species, including two new species, were identified. They are described in this paper, and a key to the species is provided.

## ﻿Material and methods

All specimens were collected by beating shrubs during the daytime or direct searching at night and are preserved in 75% ethanol. The specimens are deposited in the Museum of Tongren University, China (**TRU**). Methods follow Mi et al. (2023).

All measurements are given in millimeters. Leg measurements are given as total length (femur, patella + tibia, metatarsus, tarsus). Abbreviations used in the text and figures are as follows: **ALE** anterior lateral eye; **AME** anterior median eye; **C** conductor; **CD** copulatory duct; **CO** copulatory opening; **E** embolus; **FD** fertilization duct; **MA** median apophysis; **MOA** median ocular area; **Pc** paracymbium; **PLE** posterior lateral eye; **PME** posterior median eye; **Sp** spermatheca; **TA** terminal apophysis; **TP** tegular projection.

## ﻿Taxonomy

### ﻿Key to species of the genus *Yaginumia*

**Table:** 

1	Male	**2**
–	Female	**4**
2	Median apophysis with projection on middle part (Fig. 6A)	***Yaginumiasia***
–	Median apophysis lacking projection on middle part (Figs 2A, 4A)	**3**
3	Median apophysis curled about 90° in ventral view (Fig. 2A)	***Y.medog* Mi & Wang, sp. nov.**
–	Median apophysis curled about 45° in ventral view (Fig. 4A)	***Y.qiong* Mi & Wang, sp. nov.**
4	Copulatory openings arcuate (Fig. 1A)	***Y.medog* Mi & Wang, sp. nov.**
–	Copulatory openings almost rounded (Figs 3A, 5A)	**5**
5	Scape heart-shaped (Fig. 5B)	***Y.sia***
–	Scape tongue-shaped (Fig. 3B)	***Y.qiong* Mi & Wang, sp. nov.**

### ﻿Family Araneidae Clerck, 1757

#### ﻿Genus Yaginumia

Taxon classificationAnimaliaAraneaeAraneidae

Archer, 1960

428F9250-9BCE-508E-BB28-1D0B5C54619F

Yaginumia Archer, 1960: 14.

##### Type species.

*Araneasia* Strand, 1906.

##### Diagnosis.

**Figure 1.**
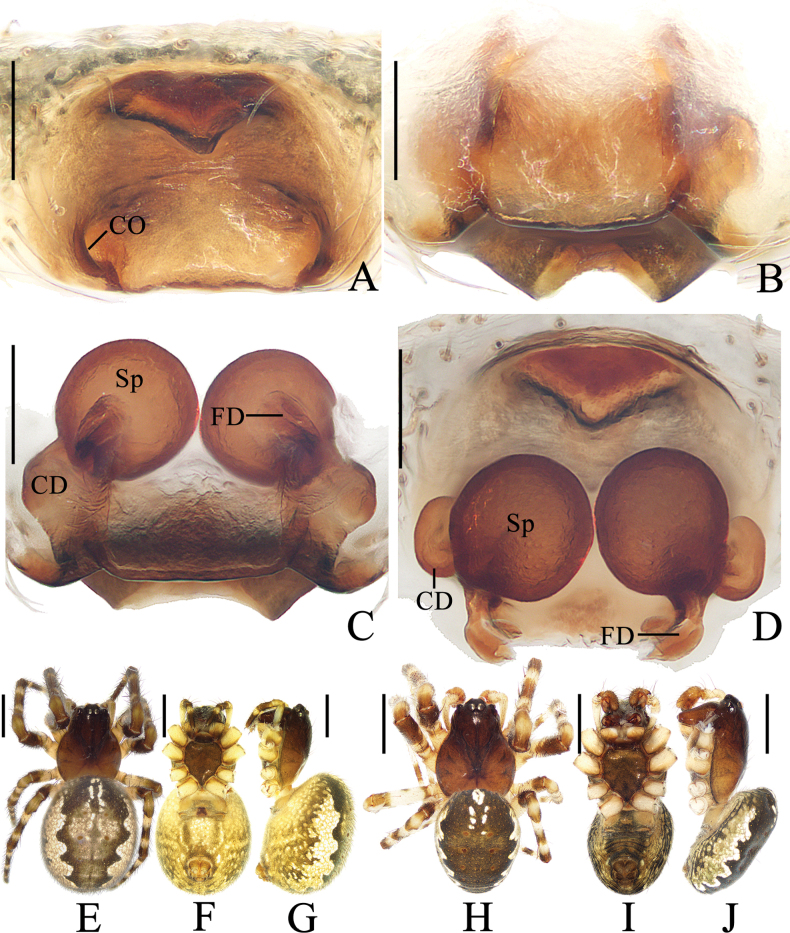
*Yaginumiamedog* Mi & Wang, sp. nov. **A–G** female paratype TRU-Araneidae-327 **H–J** male holotype **A** epigyne, ventral view **B** ibid., posterior view **C** vulva, posterior view **D** ibid., dorsal view **E, H** habitus, dorsal view **F, I** ibid., ventral view **G, J** ibid., lateral view. Abbreviations: CD copulatory duct, CO copulatory opening, FD fertilization duct, Sp spermatheca. Scale bars: 0.1 mm (**A–D**); 1.0 mm (**E–J**).

**Figure 2.**
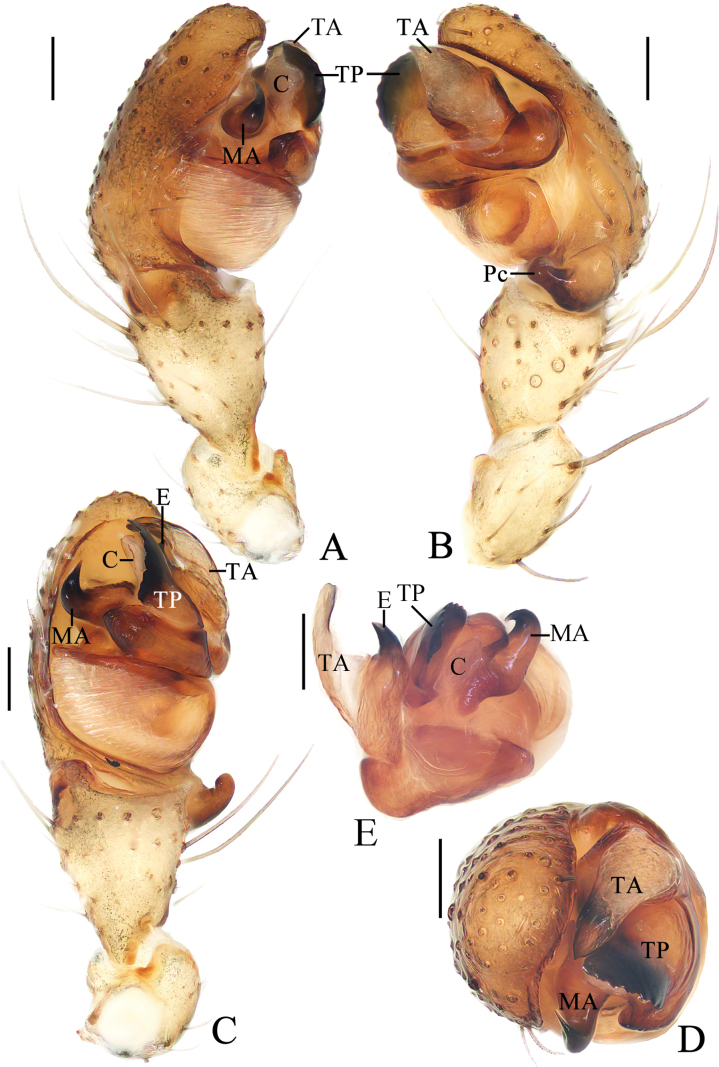
*Yaginumiamedog* Mi & Wang, sp. nov. male holotype **A** pedipalp, prolateral view **B** ibid., retrolateral view **C** ibid., ventral view **D** ibid., apical view **E** part of expanded bulb. Scale bars: 0.1 mm. Abbreviations: C conductor, E embolus, MA median apophysis, Pc paracymbium, TA terminal apophysis, TP tegular projection.

**Figure 3.**
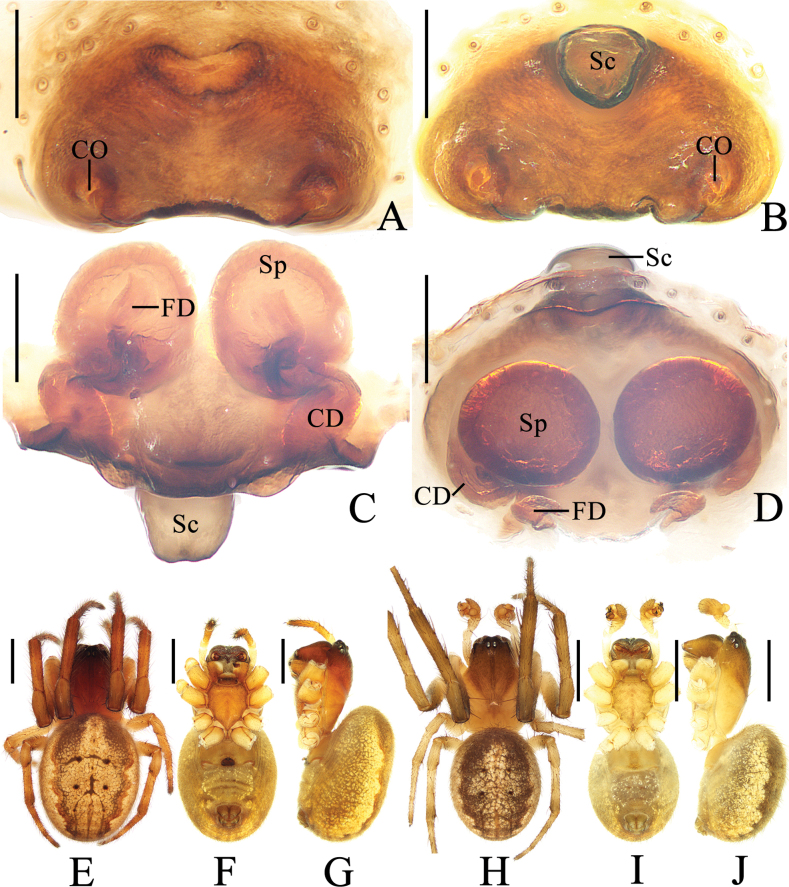
*Yaginumiaqiong* Mi & Wang, sp. nov. **A** female paratype TRU-Araneidae-330 **B–G** female paratype TRU-Araneidae-341 **H–J** male holotype **A** epigyne (scape torn off), ventral view **B** ibid, ventral view **C** vulva, posterior view **D** ibid, dorsal view **E, H** habitus, dorsal view **F, I** ibid., ventral view **G, J** ibid., lateral view. Abbreviations: CD copulatory duct, CO copulatory opening, FD fertilization duct, Sc scape, Sp spermatheca. Scale bars: 0.1 mm (**A–D**); 1.0 mm (**E–J**).

**Figure 4.**
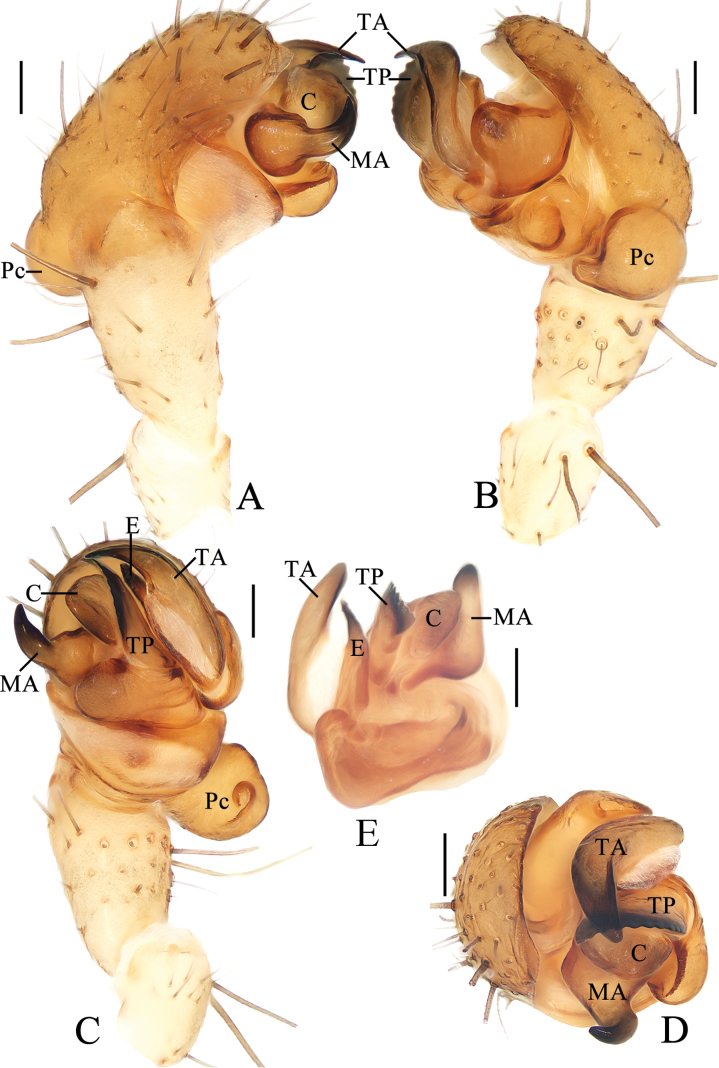
*Yaginumiaqiong* Mi & Wang, sp. nov. male holotype **A** pedipalp, prolateral view **B** ibid., retrolateral view **C** ibid., ventral view **D** ibid., apical view **E** part of expanded bulb. Abbreviations: C conductor, E embolus, MA median apophysis, Pc paracymbium, TA terminal apophysis, TP tegular projection. Scale bars: 0.1 mm.

**Figure 5.**
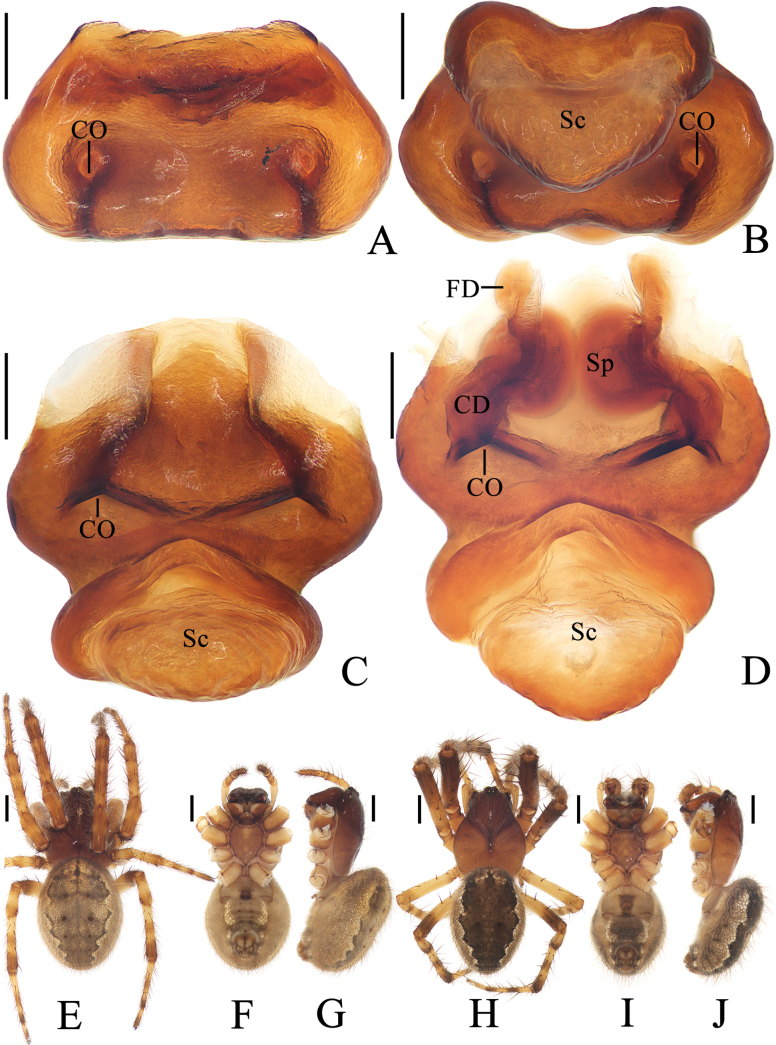
*Yaginumiasia* (Strand, 1906) **A**TRU-Araneidae-351 **B–G**TRU-Araneidae-352 **H–J**TRU-Araneidae-350 **A** epigyne (scape torn off), ventral view **B** ibid, ventral view **C** ibid., posterior view **D** vulva, posterior view **E, H** habitus, dorsal view **F, I** ibid., ventral view **G, J** ibid., lateral view. Abbreviations: CD copulatory duct, CO copulatory opening, FD fertilization duct, Sc scape, Sp spermatheca. Scale bars: 0.1 mm (**A–D**); 1.0 mm (**E–J**).

**Figure 6.**
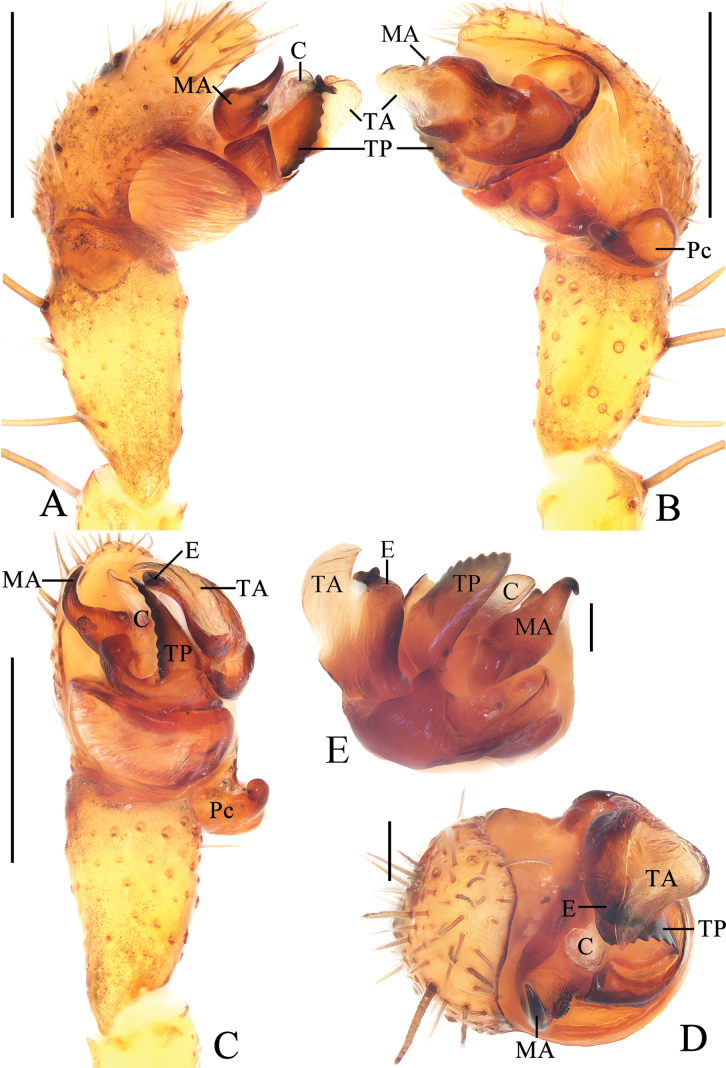
*Yaginumiasia* (Strand, 1906) TRU-Araneidae-350 **A** pedipalp, prolateral view **B** ibid., retrolateral view **C** ibid., ventral view **D** ibid., apical view **E** part of expanded bulb. Abbreviations: C conductor, E embolus, MA median apophysis, Pc paracymbium, TA terminal apophysis, TP tegular projection. Scale bars: 0.5 mm (**A–C**), 0.1 mm (**D, E**).

*Yaginumia* resembles *Guizygiella* and *Zygiella* in having a dorsoventrally flattened elliptical abdomen with almost symmetrical dorsal folium, and, in males, an enlarged paracymbium. It can be distinguished from *Guizygiella* by: 1) with triangular, toothed tegular projection (Figs 2A–E, 4A–E, 6A–E) vs absent (Zhu et al. 2003: fig. 20I, J); 2) tibia of pedipalp at least 1.5 × longer than wide in ventral view (Figs 2C, 4C, 6C) vs about equal in length and width (Zhu et al. 2003: fig. 20I); 3) epigyne with a scape (Figs 1A–D, 3A–D, 5A–D) vs lacking (Zhu et al. 2003: fig. 20F); and 4) abdomen bearing dense setae (Figs 1E–J, 3E–J, 5E–J) vs sparse setae (Zhu et al. 2003: fig. 20A). It differs from *Zygiella* by: 1) pedipalp of male with two patellar bristles (2A, B, 4A, B, 6A, B) vs only one patellar bristle (Levi 1974: 271); 2) long axis of tegulum in “horizontal” position in ventral view (Figs 2C, 4C, 6C) vs in “vertical” position (Levi 1974: figs 28, 29); 3) distance of PME–PLE is at least 3.6 × to that of PME–PME (Figs 1E, H, 3E, H, 5E, H) vs posterior eyes almost equal separated (Levi 1974: figs 26, 57); 4) abdomen bearing dense setae (Figs 1E–J, 3E–J, 5E–J) vs sparse setae (Levi 1974: fig. 26); and 5) web complete vs with a vacant sector.

##### Description.

Medium spiders with female total length of 4.25–13.10 and male total length of 3.15–8.20. Carapace pear-shaped, yellow to dark brown, darker in cephalic region than in thoracic region, fovea transverse. Endites wider than long. Labium triangular, swollen. Sternum cordiform. Legs yellow, always with dark annuli (except *Y.medog* sp. nov.). Abdomen elliptical, dorsum bearing dense setae, with dark median longitudinal folium. Ventral abdomen yellow to yellowish-gray with pale line on each side.

Pedipalp of male with two patellar bristles; tibia at least 1.5 × longer than wide; paracybium enlarged at base with small distal lobe; tegular projection almost triangular in ventral view, with toothed inner edge; median apophysis tapered distally; embolus short and slightly curved, almost totally covered by terminal apophysis; conductor membranous or weakly sclerotized; terminal apophysis prominent, weakly sclerotized, curved distally in ventral view.

Epigyne heavily sclerotized, wider than long in ventral view, with scape, the scape always torn off; copulatory openings situated on ventral surface; copulatory ducts twisted, about equal length to spermathecal diameter; spermathecae rounded, touching or nearly touching.

##### Composition.

*Yaginumiamedog* Mi & Wang, sp. nov., *Y.qiong* Mi & Wang, sp. nov. and *Y.sia* (Strand, 1906) (type species).

##### Distribution.

East Asia (China, Japan, Korea).

#### ﻿ Yaginumia medog

Taxon classificationAnimaliaAraneaeAraneidae

Mi & Wang sp. nov.

5A81C536-6DDB-5D01-B54F-0ACF603746D9

https://zoobank.org/5c95f73b-85c3-4919-8ffa-0231534a89ef

Figs 1 , 2 , 7

##### Type material.

***Holotype***: China • ♂; Xizang Autonomous Region, Medog County, Beibeng Township, De’ergong Village; 29°10.84'N, 95°8.67'E; ca 1670 m elev.; 25.V.2024; X.Q. Mi et al. leg.; TRU-Araneidae-326. ***Paratypes***: 2♀♀; same data as for holotype; TRU-Araneidae-327–328.

##### Etymology.

The species name is a noun derived from the type locality: Medog County.

##### Diagnosis.

The new species resembles *Y.qiong* sp. nov. in appearance and genitalia structures, but it can be distinguished as follows: 1) median apophysis strongly curled about 90° in ventral view (Fig. 2C) vs slightly curled about 45° (Fig. 4C); 2) basal part of paracybium ~2.1 × wider than distal part in retrolateral view (Fig. 2B) vs ~3.4 × wider (Fig. 4B); 3) copulatory openings arcuate (Fig. 1A) vs almost rounded (Fig. 3A); 4) spermathecae touching to each other (Fig. 1C, D) vs separated (Fig. 3C, D); and 5) legs with annuli (Fig. 1E, H) vs lacking (Fig. 3E, H).

##### Description.

**Male** (holotype, Figs 1H–J, 2). Total length 3.20. Carapace 1.85 long, 1.35 wide. Abdomen 2.10 long, 1.45 wide. Clypeus 0.05 high. Eye sizes and interdistances: AME 0.13, ALE 0.08, PME 0.10, PLE 0.08, AME–AME 0.10, AME–ALE 0.10, PME–PME 0.05, PME–PLE 0.18, MOA length 0.30, anterior width 0.33, posterior width 0.25. Leg measurements: I 6.65 (1.85, 2.40, 1.70, 0.70), II 5.70 (1.60, 2.05, 1.40, 0.65), III 3.50 (1.10, 1.10, 0.75, 0.55), IV 4.55 (1.40, 1.55, 1.10, 0.50). Carapace dark brown in cephalic region and yellowish-brown in thoracic region. Cervical groove conspicuous. Chelicerae dark brown, with four promarginal and three retromarginal teeth. Endites and labium dark brown at base, with paler tip. Sternum dark brown. Legs yellow with brown annuli. Abdomen ~1.45 × longer than wide, dorsal folium extended from anterior to posterior, grayish-brown with white spots anteriorly. Venter abdomen yellow to yellowish-brown. Spinnerets yellowish-brown.

***Pedipalp*** (Fig. 2): tibia ~1.78 × longer than wide in ventral view; paracybium enlarged at base and lobe-like distally; tegular projection triangular, with toothed inner edge; median apophysis tapered and curled about 90° distally in ventral view; embolus almost straight, shorter than conductor; conductor membranous; terminal apophysis weakly sclerotized, covering embolus.

**Female** (paratype TRU-Araneidae-327, Fig. 1A–G). Total length 4.55. Carapace 2.10 long, 1.55 wide. Abdomen 2.90 long, 2.30 wide. Clypeus 0.05 high. Eye sizes and interdistances: AME 0.13, ALE 0.08, PME 0.13, PLE 0.08, AME–AME 0.13, AME–ALE 0.08, PME–PME 0.05, PME–PLE 0.20, MOA length 0.33, anterior width 0.35, posterior width 0.28. Leg measurements: I 6.75 (2.00, 2.45, 1.55, 0.75), II 5.80 (1.75, 2.05, 1.30, 0.70), III 3.85 (1.25, 1.30, 0.75, 0.55), IV 5.30 (1.70, 1.85, 1.15, 0.60). Habitus similar to that of male.

***Epigyne*** (Fig. 1A–D): ~1.57 × wider than long in ventral view; copulatory openings arcuate, situated on ventral surface; copulatory ducts twisted, about equal length to spermathecal diameter; spermathecae rounded, touching.

##### Variation.

Total length: ♀♀ 3.15–3.30 (*N* = 2).

##### Distribution.

China (Xizang).

##### Comment.

Judging from the broken vestige, we conclude it must have an epigynal scape.

#### ﻿ Yaginumia qiong

Taxon classificationAnimaliaAraneaeAraneidae

Mi & Wang sp. nov.

16DE4DEC-C482-5E71-9F2A-4A01F691D6A6

https://zoobank.org/FC336132-DB32-4363-BB2D-D6F5851CEA47

Figs 3 , 4 , 7

##### Type material.

***Holotype***: China – Hainan Province, • ♂; Lingshui Li Autonomous County, Diaoluoshan National Nature Reserve, Popular Science Base; 18°40.25'N, 109°53.66'E; ca 260 m elev.; 26.VII.2023; C. Wang et al. leg.; TRU-Araneidae-329. ***Paratypes***: 10♀♀; same data as for holotype; TRU-Araneidae-330–339 • 1♂; Ledong Li Autonomous County, Jianfeng Township, Jianfengling National Nature Reserve, Tianchi; 18°44.45'N, 108°51.49'E; ca 860 m elev.; 11.IV.2019; C. Wang & Y.F. Yang leg.; TRU-Araneidae-340 • 1♀; Ledong Li Autonomous County, Jianfeng Township, Jianfengling National Nature Reserve, Yulingu; 18°44.79'N, 108°55.76'E; ca 630 m elev.; 15.IV.2019; C. Wang & Y.F. Yang leg.; TRU-Araneidae-341 • 2♀♀; Ledong Li Autonomous County, Jianfeng Township, Jianfengling National Nature Reserve, Tianchi; 18°44.82'N, 108°51.64'E; ca 810 m elev.; 15.IV.2019; C. Wang & Y.F. Yang leg.; TRU-Araneidae-342–343.

##### Etymology.

The species name is a noun in apposition derived from Chinese pinyin qiong, short name of the type locality, Hainan.

##### Diagnosis.

See diagnosis of *Y.medog* Mi & Wang, sp. nov.

##### Description.

**Male** (holotype, Figs 3H–J, 4). Total length 3.95. Carapace 2.00 long, 1.50 wide. Abdomen 2.25 long, 1.80 wide. Clypeus 0.05 high. Eye sizes and interdistances: AME 0.13, ALE 0.08, PME 0.10, PLE 0.08, AME–AME 0.13, AME–ALE 0.10, PME–PME 0.04, PME–PLE 0.23, MOA length 0.33, anterior width 0.38, posterior width 0.24. Leg measurements: I 6.70 (1.90, 2.45, 1.60, 0.75), II 5.90 (1.75, 2.05, 1.40, 0.70), III 3.60 (1.15, 1.15, 0.80, 0.50), IV 4.65 (1.40, 1.60, 1.10, 0.55). Carapace red brown in cephalic region and yellow in thoracic region. Cervical groove conspicuous. Chelicerae yellowish-brown, with five promarginal and three retromarginal teeth. Endites and sternum yellow. Labium yellow at base, with paler tip. Legs yellow to grayish-yellow, without annuli. Abdomen ~1.25 × longer than wide, dorsum grayish-brown with paler middle patch. Venter abdomen grayish-yellow. Spinnerets grayish-yellow.

***Pedipalp*** (Fig. 4): tibia ~1.5 × longer than wide; paracybium extremely enlarged into disc, lobe-like distally; tegular projection triangular, with toothed inner edge; median apophysis rounded at base, tapered and curled distally; embolus almost straight, shorter than conductor; conductor weakly sclerotized, rounded distally; terminal apophysis longer than tegular projection, curved to C-shape in apical view.

**Female** (paratype TRU-Araneidae-330, Fig. 3A, paratype TRU-Araneidae-341, Fig. 3B–G). Total length 4.55. Carapace 2.20 long, 1.75 wide. Abdomen 3.20 long, 2.40 wide. Clypeus 0.08 high. Eye sizes and interdistances: AME 0.13, ALE 0.08, PME 0.10, PLE 0.08, AME–AME 0.13, AME–ALE 0.13, PME–PME 0.05, PME–PLE 0.25, MOA length 0.30, anterior width 0.38, posterior width 0.25. Leg measurements: I 6.50 (1.90, 2.40, 1.55, 0.65), II 5.75 (1.70, 2.10, 1.35, 0.60), III 3.70 (1.15, 1.25, 0.80, 0.50), IV 5.05 (1.65, 1.80, 1.10, 0.50). Habitus similar to that of male.

***Epigyne*** (Fig. 3A–D): ~1.71 × wider than long in ventral view, with short, tongue-shaped scape; copulatory openings rounded in ventral view, situated on ventral surface; copulatory ducts twisted, about equal length to spermathecal diameter; spermathecae rounded, nearly touching.

##### Variation.

Total length: ♂♂ 3.85–3.95 (*N* = 2); ♀♀ 4.25–5.70 (*N* = 13).

##### Distribution.

China (Hainan).

#### ﻿ Yaginumia sia

Taxon classificationAnimaliaAraneaeAraneidae

(Strand, 1906)

43BB8BBB-7B66-56EB-9267-AE233AD071A8

Figs 5 , 6 , 7

Aranea sia Strand, in Bösenberg and Strand 1906: 237, pl. 4, fig. 24 (♀); Song 1980: 94, fig. 40a–d (♂♀) (type material in Senckenberg Museum, Frankfurt am Main (SMF), Germany, not examined).
Araneus sia Yaginuma, 1960: 115, fig. 50.1–3; Lee 1966: 40, fig. 10m–o; Yaginuma 1971: 54, fig. 50.1–3 (♂♀); Yin 1978: 2, fig. 3A–D (♂♀); Hu 1984: 97, fig. 92.1–2 (♂♀); Zhao 1993: 233, fig. 107a–b (♂♀); Zhao 1995: 950, fig. 446a–b (♂♀).
Yaginumia sia Archer, 1960: 14, figs 1–4 (♂♀); Namkung et al. 1972: 93, fig. 11 (♀); Guo 1985: 75, fig. 2–27.1–3 (♂♀); Yaginuma 1986: 109, fig. 57.6 (♂♀); Zhang 1987: 89, fig. 73.1–4 (♂♀); Ishinoda 1989: 21, figs 12, 13 (♂♀); Chikuni 1989: 75, fig. 39 (♂♀); Feng 1990: 101, fig. 76.1–3 (♂♀); Chen and Gao 1990: 73, fig. 92a–c (♂♀); Chen and Zhang 1991: 91, fig. 82.1–3 (♂♀); Zhao 1993: 262, fig. 123a, b (♂♀); Zhao 1995: 979, fig. 462a, b (♂♀); Yin et al. 1997: 395, fig. 286a–d (♂♀); Song et al. 1999: 309, figs 184G–I, 185L (♂♀); Namkung 2002: 303, fig. 19.64a, b (♂♀); Kim and Kim 2002: 225, figs 69, 155, 288, 289 (♂♀); Kim and Cho 2002: 270, figs 685–690 (♀); Namkung 2003: 305, fig. 19.64a, b (♂♀); Tanikawa 2007: 93, figs 299, 300, 760–763 (♂♀); Tanikawa 2009: 463, figs 331–334 (♂♀); Zhu and Zhang 2011: 248, fig. 179A–D (♂♀); Yin et al. 2012: 762, fig. 381a–e (♂♀); Kim and Lee 2012: 109, fig. 81A–C, plate 24 (♂♀); Baba and Tanikawa 2015: 69, 4 fig. (♂♀).

##### Material examined.

China – Guizhou Province • 2♂♂4♀♀; Tongren City, Shiqian County, Pingshan Township, Fodingshan Village, Yaoshang; 27°20.54'N, 108°9.50'E; ca 640 m elev.; 11.VII.2017; X.Q. Mi et al. leg.; TRU-Araneidae-344–349 • 2♂♂24♀♀; Tongren City, Bijiang District, Chuangdong Township, around campus of Tongren University; 27°46.66'N, 109°13.00'E; ca 570 m elev.; 7–15.VII.2017; L.F. Chen et al. leg.; TRU-Araneidae-350–375 • 3♂♂10♀♀; Qiandongnan Miao and Dong Autonomous Prefecture, Shibing County, Baiduo Township, Baiduo Village, Heichong; 27°9.37'N, 108°7.40'E; ca 980 m elev., 20.VII.2019; X.Q. Mi et al. leg.; TRU-Araneidae-376–388 • 4♂♂2♀♀; Zunyi City, Huichuan District, Banqiao Township, Loushanguan Village; 27°58.06'N, 106°53.20'E; ca 920 m elev.; 17.VII.2024; X.Q. Mi leg.; TRU-Araneidae-389–394. – Hubei Province • 4♂♂12♀♀; Wuhan City, Hongshan District, Ma’anshan Forest Park; 30°31.65'N, 114°26.12'E; ca 30 m elev.; 3.VI.2018; X.Q. Mi leg.; TRU-Araneidae-395–410.

##### Diagnosis.

This species resembles *Y.medog* Mi & Wang, sp. nov. in appearance and genitalia structures, but it can be distinguished as follows: 1) median apophysis with a projection on middle part (Fig. 6A, C–E) vs lacking (Fig. 2A, C–E); 2) median apophysis curled about 45° in ventral view (Fig. 6C) vs about 90° (Fig. 2C); 3) copulatory openings almost rounded in ventral view (Fig. 5A, B) vs arcuate (Fig. 1A); and 4) diameter of spermathecae about 1/4 width of epigynal base (Fig. 5D) vs about 2/5 width of epigynal base (Fig. 1D).

**Figure 7.**
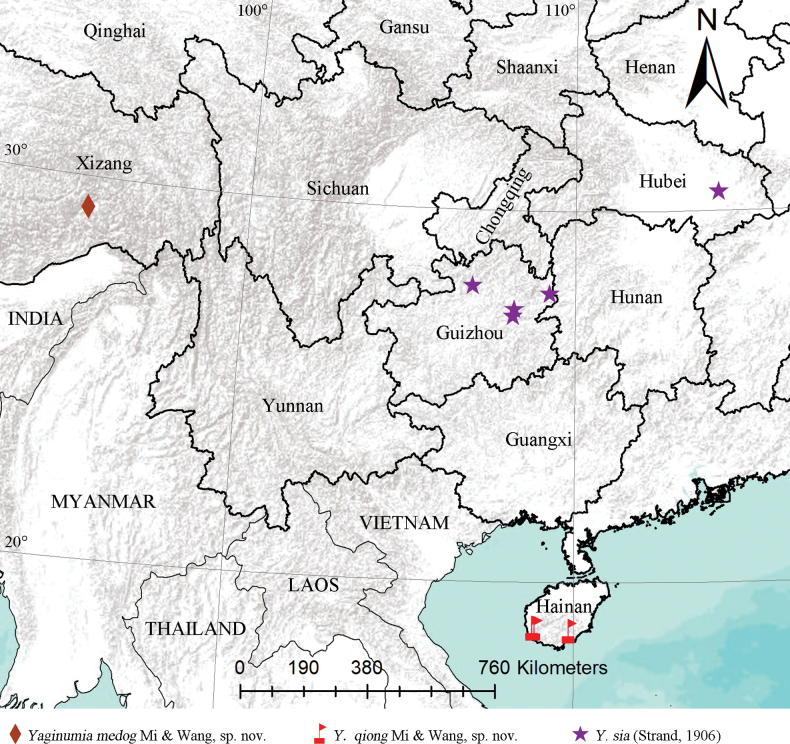
Distribution in China of the examined specimens of *Yaginumia*.

##### Description.

**Male** (TRU-Araneidae-350, Figs 5H–J, 6). Total length 6.30. Carapace 3.30 long, 2.50 wide. Abdomen 3.70 long, 2.60 wide. Clypeus 0.08 high. Eye sizes and interdistances: AME 0.18, ALE 0.10, PME 0.13, PLE 0.10, AME–AME 0.15, AME–ALE 0.28, PME–PME 0.05, PME–PLE 0.45, MOA length 0.48, anterior width 0.48, posterior width 0.30. Leg measurements: I 13.30 (3.70, 5.10, 3.20, 1.30), II 11.60 (3.20, 4.20, 3.00, 1.20), III 6.50 (2.10, 2.10, 1.50, 0.80), IV 9.10 (2.70, 3.30, 2.20, 0.90). Carapace dark brown in cephalic region and yellowish-brown in thoracic region. Cervical groove conspicuous. Chelicerae dark brown, with four promarginal and three retromarginal teeth. Endites and labium dark brown at base, with paler tip. Sternum yellowish-brown. Legs yellow with brown annuli. Abdomen ~1.42 × longer than wide, dorsal grayish-brown with paler edges. Venter abdomen grayish-yellow with pair of lateral white patches. Spinnerets grayish-yellow.

***Pedipalp*** (Fig. 6): tibia ~1.96 × longer than wide in ventral view; paracybium enlarged at base, lobe-like distally; tegular projection triangular, with serrated edge; median apophysis wide at base, with projection at middle part, tapered and curled distally; embolus slightly curled, shorter than conductor; conductor membranous, longer than wide; terminal apophysis with weakly sclerotized distal portion, about equal length to tegular projection.

**Female** (TRU-Araneidae-351, Fig. 5A, TRU-Araneidae-352, Fig. 5B–G). Total length 7.00. Carapace 3.00 long, 2.80 wide. Abdomen 4.30 long, 3.30 wide. Clypeus 0.08 high. Eye sizes and interdistances: AME 0.18, ALE 0.10, PME 0.13, PLE 0.10, AME–AME 0.15, AME–ALE 0.25, PME–PME 0.05, PME–PLE 0.45, MOA length 0.45, anterior width 0.45, posterior width 0.30. Leg measurements: I 11.90 (3.40, 4.40, 3.00, 1.10), II 10.30 (3.00, 3.80, 2.50, 1.00), III 6.20 (2.00, 2.20, 1.30, 0.70), IV 8.90 (2.80, 3.20, 1.90, 1.00). Habitus similar to that of male.

***Epigyne*** (Fig. 5A–D): ~1.55 × wider than long in ventral view, with heart-shaped scape; copulatory openings rounded, situated on ventral surface; copulatory ducts twisted, a bit longer than spermathecal diameter; spermathecae rounded, touching.

##### Variation.

Total length: ♂♂ 5.60–8.20 (*N* = 15); ♀♀ 5.80–13.10 (*N* = 52). Scape torn off in most individuals.

##### Distribution.

China (Anhui, Fujian, Guangdong, Guangxi, Guizhou, Henan, Hubei, Hunan, Jiangsu, Shaanxi, Shandong, Sichuan, Taiwan, Xinjiang, Zhejiang), Korea, Japan.

## ﻿Discussion

According to the literature and our fieldwork experience, *Y.sia* shows a high propensity to live close to humans; the specimens were collected in houses, under the eaves, around bridges, rice fields and cotton fields; it is widely distributed in eastern Asia, human activities may influence its distribution. However, the two new *Yaginumia* species were collected from low shrubs and didn’t show the propensity to live close to humans.

The edges of the pedipalpal tegulum of most araneids are rounded and almost smooth, or with low ridges in some species; a long tegular process is rarely found in araneids. Levi (1974) introduced the term “projection of tegulum” for *Y.sia* for the first time; although the long tegular projection looks like a conductor, it is an extension of the tegulum, and the conductor is a structure of the outer bulb close to the tegulum. A long tegular projection was also described in some Chinese araneids species, such as *Hypsosingaalboria* Yin, Wang, Xie & Peng, 1990, *H.sanguinea* (C. L. Koch, 1844), *Lariniaastrigera* Yin, Wang, Xie & Peng, 1990, *L.cyclera* Yin, Wang, Xie & Peng, 1990, *L.dinanea* Yin, Wang, Xie & Peng, 1990, *L.elegans* Spassky, 1939, *L.nolabelia* Yin, Wang, Xie & Peng, 1990, *L.phthisica* (L. Koch, 1871), *L.wenshanensis* Yin & Yan, 1994, and *Lariniariaargiopiformis* (Bösenberg & Strand, 1906).

## Supplementary Material

XML Treatment for Yaginumia

XML Treatment for Yaginumia medog

XML Treatment for Yaginumia qiong

XML Treatment for Yaginumia sia

## ﻿Additional information

### Author ORCIDs

Xiaoqi Mi https://orcid.org/0000-0003-1744-3855

Cheng Wang https://orcid.org/0000-0003-1831-0579

### Data availability

All of the data that support the findings of this study are available in the main text.
